# Real-Time Dose-Guided Radiation Therapy

**DOI:** 10.1016/j.ijrobp.2025.04.019

**Published:** 2025-05-06

**Authors:** Paul J. Keall, Issam El Naqa, Martin F. Fast, Emily A. Hewson, Nicholas Hindley, Per Poulsen, Chandrima Sengupta, Neelam Tyagi, David E.J. Waddington

**Affiliations:** a Image X Institute, University of Sydney, Sydney, Australia; b Department of Machine Learning, Moffitt Cancer Center, Tampa, Florida; c Department of Radiotherapy, University Medical Center Utrecht, Utrecht, The Netherlands; d Danish Centre for Particle Therapy, Aarhus University Hospital, Aarhus, Denmark; e Department of Clinical Medicine, Aarhus University, Aarhus, Denmark; f Department of Medical Physics, Memorial Sloan Kettering Cancer Center, New York, New York

## Abstract

Dramatic strides have been made in real-time adaptive radiation therapy, where treating single tumors as dynamic but rigid bodies has demonstrated a halving of toxicities for prostate cancer. However, the human body is much more complex than a rigid body. This review explores the ongoing development and future potential of dose-guided radiation therapy, where the three core process steps of volumetric imaging of the patient, dose accumulation, and dose-guided treatment adaptation occur quasi-continuously during treatment, fully accounting for the complexity of the dynamic human body.

The clinical evidence supporting real-time adaptive radiation therapy was reviewed. The foundational studies, status, and potential of real-time volumetric imaging using both x-ray and magnetic resonance imaging technology were described. The development of real-time dose accumulation to the dynamic patient was evaluated, and a method to measure real-time dose delivery was assessed. The growth of real-time treatment adaptation was examined.

Literature demonstrates continued improvements in patient outcomes because the treatment becomes more conformal to the dynamic patient. Real-time volumetric imaging using both x-ray and magnetic resonance imaging technology is poised for broader implementation. Real-time dose accumulation has demonstrated clinical feasibility, with approximations made to achieve real-time operation. Real-time treatment adaptation to deforming targets and multiple targets has been experimentally demonstrated. Tying together the inputs of the real-time volumetric anatomy and dose accumulation is real-time treatment adaptation that uses the available degrees of freedom to optimize the dose delivered to the patient, maximizing the treatment intent. Opportunities exist for artificial intelligence to accelerate the application of dose-guided radiation therapy to broader patient use.

In summary, the emerging field of real-time dose-guided radiation therapy has the potential to significantly improve patient outcomes. The advances are primarily software-driven and therefore could be widely available and cost-effective upgrades to improve imaging and targeting cancer.

## Introduction

**Fig. 1.**
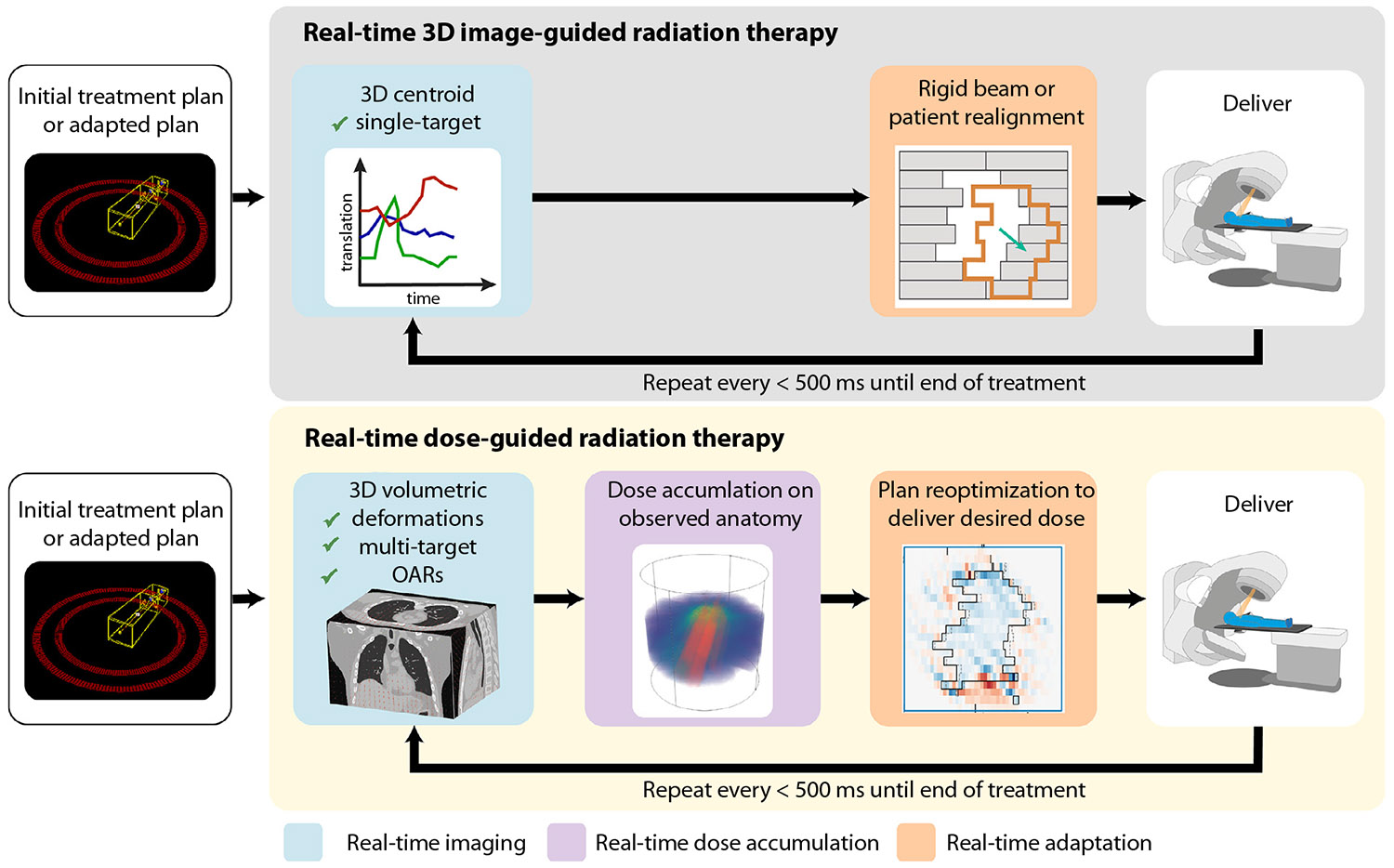
A comparison of real-time 3-dimensional (3D) image guided radiation therapy with real-time dose-guided radiation therapy. The key differences are moving from a point, for example, the target centroid, to a full volumetric representation of the patient in each stage and the change in focus from anatomy to dose because the dose is more closely tied to patient outcomes. Abbreviation: OAR = organ at risk.

In 2017, this journal published a review of real-time 3-dimensional (3D) image guided radiation therapy (IGRT).^1^ Substantial progress has been made in the scientific development and clinical implementation of real-time 3D IGRT with demonstrated improved patient outcomes.^2,3^ Real-time 3D IGRT is limited to aligning the treatment beam to a single rigid target in the human body. Emerging from the successes of real-time 3D IGRT, through rapid advances in software and hardware in parallel with the development of new technology, is real-time dose-guided radiation therapy. Real-time dose-guided radiation therapy is defined as the process where volumetric imaging of the patient, dose accumulation, and dose-guided treatment adaptation occur quasi-continuously during treatment to fully account for the complexity of the dynamic human body. Because deformable image registration (DIR) is typically required for volumetric imaging of the patient, we include multitarget tracking and simultaneous target and organ at risk (OAR) tracking as stepping-stone implementations of real-time dose-guided radiation therapy, toward the goal of full dose-guided radiation therapy. A comparison of real-time 3D IGRT with real-time dose-guided radiation therapy is shown in Figure 1. Throughout this review, the term “real-time” is used. The American Association of Physicists in Medicine Task Group 264 defines “real-time” as occurring within 500 ms.^4^

This article (1) reviews the clinical drivers for 3D IGRT and real-time dose-guided radiation therapy and (2) describes the scientific development, state of the art, and future prospects for real-time dose-guided radiation therapy for the three core process steps of real-time volumetric imaging, dose accumulation, and dose-guided treatment adaptation.

## Clinical Drivers for 3-Dimensional IGRT and Real-Time Dose-Guided Radiation Therapy

Tumor motion during radiation therapy leads to higher healthy tissue doses, reduced tumor doses, and poorer treatment outcomes, particularly for highly mobile tumors near radiosensitive organs. SABR, whether multiple or a single fraction,^5^ requires accurate dose delivery because any miss in a single fraction would have a more significant impact and cannot be easily compensated for the following fractions. The dosimetric impact of motion includes underdose in 18% of prostate treatments,^6^ a 30% increase in the irradiated lung volume for patients with lung cancer,^7^ and a lower prescription dose for 55% of patients with liver cancer.^8^

Besides rigid motion, tumors, involved lymph nodes and nearby organs in the prostate, lung, pancreas, and liver are subject to large differential motion^9,10^ and deformations.^11-15^ Such complex motion is not well-described by rigid real-time 3D IGRT methods and challenges the localization of the tumors and OARs. Instead, real-time volumetric imaging aims to capture the 3D motion and deformation of the tumor and surrounding organs, with the goal of allowing more precise targeting, dose accumulation, and adaptation.^16,17^

Recent studies and clinical trials have demonstrated that cancer targeting with more accurate and frequent imaging restores the planned dose distribution, improves local control, and reduces treatment-related toxicity as discussed in the following sections.

## Clinical drivers for prostate tumors

Prostate motion^6^ and deformation^12^ during treatment are generally slow and small, governed by bladder, bowel, and skeletal motion, but could be as large as 1.5 cm, with increasing risks of recurrence and toxicity leading to urinary and bowel dys-function if uncorrected.^18,19^ The dosimetric driver for real-time 3D IGRT for prostate was highlighted by Lovelock et al^20^ (electromagnetic transponder-guided gating) and Keall et al^6^ (marker-guided gating or multileaf collimator [MLC] tracking) where patients treated with real-time 3D IGRT had clinically significant superior dose coverage compared with treatment without real-time IGRT. Patients with prostate cancer treated with real-time 3D IGRT using electromagnetic transponders had significantly lower bowel morbidity and improved health-related quality of life compared with a cohort treated without real-time IGRT.^21^ In the MRI-guided stereotactic body radiotherapy for prostate cancer (MIRAGE) trial randomizing daily (computed tomography [CT] guidance) versus real-time 3D IGRT (magnetic resonance imaging [MRI]-Linac-guided beam hold), aggressive margin reduction enabled by real-time IGRT demonstrated reduction in physician-scored grade ≥2 acute genitourinary toxicity from 47% to 22%, 100% reduction in grade ≥2 acute gastrointestinal toxicity, and reduced decrements in the patient-reported quality of life.^2^ Further analysis of the MIRAGE trial data demonstrated that treatment fractions with large intrafraction motion were associated with increased toxicity, indicating the importance of real-time IGRT to improve outcomes in prostate radiation therapy.^22^ On standard radiation therapy systems, a real-time 3D IGRT system (kilovoltage-Megavoltage [kV-MV] triangulation-guided gating) reduced grade ≥2 urinary toxicity in patients compared with treatments using a real-time 2-dimensional (2D) IGRT system (2D kilovoltage [kV]-guided gating), (11% vs 20%),^3^ indicating that real-time 3D IGRT positively impacts clinical outcomes with improved motion management. Together, these studies provide direct evidence that increased toxicity was associated with large intrafraction motion, and patient toxicity was halved when real-time IGRT was enabled. These results emphasize the critical role of improved real-time image guidance methods to effectively mitigate prostate intra-fraction motion.

## Clinical drivers for abdominal and thoracic tumors

Tumors in the lung, liver, pancreas, esophagus and surrounding radiosensitive organs, such as the heart, duodenum and stomach move up to 5 cm with respiration and other physiological motion and may undergo significant volume changes during radiation therapy.^11,13-15,23^

In lung SABR, intrafraction motion and baseline changes are accounted for by applying large treatment margins. Large margins result in the increased irradiation of healthy tissues and restrict isotoxic target dose escalation.^7,24,25^ Although every 1-gray (Gy) increase in lung tumor dose results in a 4% improvement in survival,^26^ every 1-Gy decrease in mean lung dose results in a 2% reduction in pneumonitis.^27^ Real-time IGRT technologies, including electromagnetic transponder-guided MLC tracking,^28^ Vero dynamic tracking,^29^ and CyberKnife Robotic Radiosurgery-guided tracking,^30^ demonstrated up to 30% reduction in planning target volume and reduction in dose to the OARs particularly for patients with large tumor motion.^7^ Planning studies using MRI guidance demonstrated margin reduction–enabling increased healthy lung tissue and OAR-sparing and isotoxic tumor dose escalation.^24,25^ However, these studies are limited to adapting to a single rigid target, whereas human anatomy is constantly deforming. Adapting to the differential motion of structures by tracking deformed anatomy may lead to reduced motion-included dose errors. Real-time dose-guided radiation therapy, therefore, may enable further shrinking of high-dose volumes to further alleviate treatment-related toxicity.

Liver SABR is particularly challenging because higher doses are associated with improved local control rate,^31^ with >132 Gy biological equivalent dose showing the greatest benefit.^32^ However, a higher dose to the healthy liver volume from larger radiation beams accounting for tumor motion increases liver toxicity,^33^ limiting the prescription dose to the tumor.^8^ The dosimetric driver of real-time 3D IGRT for liver SABR was highlighted in recent studies, where patients treated with real-time marker-guided gating on standard radiation therapy systems had clinically significant superior dose coverage compared with treatments without real-time IGRT (delivered dose error to 100% of the gross tumor volume: 2% vs 10%).^34,35^ Real-time IGRT with MRI-Linacs has demonstrated decreased healthy liver dose^36^ and reduced amount of irradiated healthy liver parenchyma (% healthy liver mean, V15 = 16.27 vs 22.76) without any decrease of tumor control rate,^37^ a finding that may enable dose escalation where needed.

Similar to liver, increasing the amount of radiation delivered to the pancreas^38-41^ and esophagus^42^ enabled by real-time 3D IGRT with marker guidance or MRI-Linac guidance may improve patient treatment outcomes and provide an option for low-toxicity and minimally invasive therapy for these patients.

## Clinical drivers for multitumor and OARs

The detrimental effect of intrafraction tumor motion can be even more severe in patients with advanced prostate cancer,^43^ non-small cell lung cancer,^44-46^ or oligometastatic cancer, when multiple targets are treated simultaneously. This is because the primary tumor and lymph nodes may undergo large differential motion leading to large dose deviations^9,10^ above clinical tolerance and overdosage of critical structures. In patients with prostate cancer, a 1-cm prostate shift may result in a 14% decrease in prostate D95 dose.^9^ When the beam is shifted to compensate for the prostate motion, the prostate receives the correct dose; however, the pelvic lymph nodes may be underdosed by 14%.^9^ For patients with lung cancer, 1 study evaluating 4-dimensional CT (4DCT) scans found that at least 1 node moved more than the primary tumor in 11 of the 16 patients.^44^ Because of the differential motion, correcting for the primary tumor position can lead to important dose deviations in nodal volumes and overdosage of critical structures in up to 13% and 20% of patients, respectively.^10^ This large differential motion observed for multitumor treatments is usually accounted for by applying larger treatment margins that lead to more dose to healthy tissues, for example, either a large rectal dose or a large bladder and small bowel dose in patients with prostate cancer.^43^

Intrafraction OAR motion adds another level of uncertainty in the radiation delivery. Multiple studies have reported changes in OAR volumes and positions with respect to the tumor over treatment fractions, suggesting larger treatment margins to be applied to account for the differential motion.^14,47-49^ A prostate cancer review showed that during treatment, seminal vesicle motion ranged up to 7 mm measured using repeated cone beam CT and MRI scans and was largely uncorrelated to prostate motion, requiring either larger treatment margins or individual intrafraction tracking of prostate and seminal vesicles.^50^ An MRI-Linac-guided study reported the intrafraction mean volume variation of 85%, 60%, and 24%, respectively, for the small bowel, the colon, and the duodenum for upper-abdominal SABR.^51^ The corresponding variation of maximum dose for the colon, small bowel, and duodenum was, respectively, about 4.3, 3.4, and 2.8 Gy. Another study investigating pancreatic cancer with MRI guidance found that because of intrafraction OAR motion, the OARs moved from the isotoxic low-dose region to the high-dose region, exceeding dose-volume constraints,^52^ indicating that measures need to be taken to mitigate the OAR intrafraction motion.

Together, the clinical data suggest that real-time dose-guided radiation therapy will be an effective solution to spare the OARs while ensuring accurate dose delivery to the tumors and escalating tumor dose where needed, thereby improving local control and mitigating treatment-related toxicity in patients.

In the next few sections, we discuss scientific development, state of the art, and future prospects for real-time dose-guided radiation therapy technologies.

## Real-Time Volumetric Imaging

Capturing dynamic human anatomic changes using real-time imaging, that is, on a timescale where anatomic changes can be mitigated using adaptive radiation therapy techniques, is challenging. Imaging during radiation therapy has traditionally relied on x-ray-based techniques. However, the emergence of MRI-Linacs represents a significant advancement, offering superior soft tissue contrast and the ability to visualize tumors and surrounding organs without ionizing radiation. Despite these benefits, both x-ray and MRI-based systems face challenges in achieving real-time volumetric imaging, which is crucial for adapting treatment to the dynamic movements of internal organs during therapy.

## Real-time x-ray volumetric imaging

Estimating volumetric images from the sparse 2D x-ray imaging data has traditionally used compressed sensing methods that leverage total variation,^53^ spatial-temporal correlations,^54^ known subregions,^55^ and L1 regularization.^56^ However, these techniques typically require multiple projection images, which take time to acquire, and may involve slow iterative optimization algorithms, making these approaches unsuitable for real-time implementation. Increasingly, deep learning (DL) methods compatible with real-time usage are being developed. Pioneering work by Shen et al^57^ pushed sparse sampling to the limit by using a convolutional neural network (CNN) to perform patient-specific 3D image reconstruction from a single 2D image. Related approaches have also leveraged generative adversarial networks^58,59^ and implicit neural representation.^60^ A common limitation of these existing DL-based volumetric imaging methods is that they require the training of a new neural network on a new dataset for changes in imaging parameters, for example, a change in imaging angle.

**Fig. 2.**
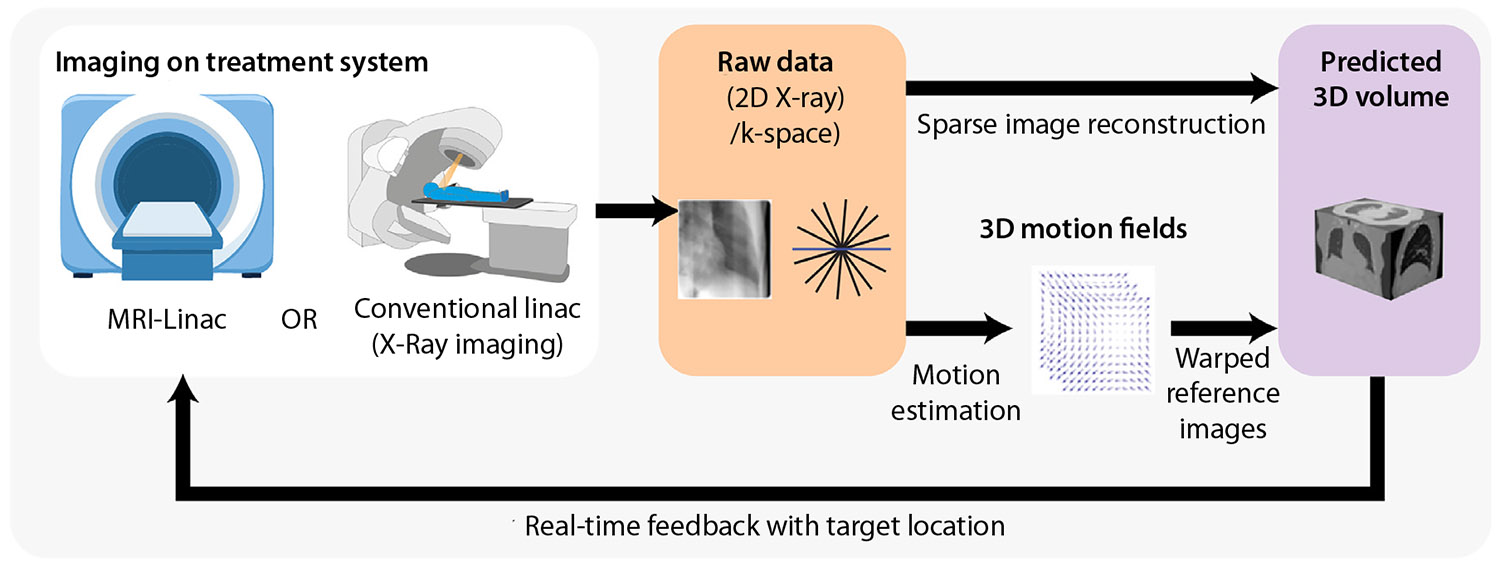
A framework for generating volumetric images from 2-dimensional (2D) projections.Two-dimensional x-ray images or k-space data are acquired in real time and used to create a 3-dimensional (3D) volume at each time point during the treatment delivery. *Abbreviation:* MRI = magnetic resonance imaging.

An alternative approach to creating new real-time volumetric images is to estimate 3D internal organ motion by warping pretreatment 3D images and contours based on online 2D images. Among the sources of intrafraction motion, respiratory-induced motion has been the most studied because of the large displacements of a centimeter or more and its broad influence across the thorax and abdomen.^61^ Seminal work by Zhang et al^62^ found that 2 principal components accounted for up to 90% of the variance of 3D deformation vector fields (DVFs) computed from 4DCT scans. Mathematically, the compressibility of respiratory motion to a few principal components reflects the strong spatial correlations of these fields. Physiologically, this relates to the inferior-anterior descent of the diaphragm during inhalation as the predominant driver of respiration. Building on this work by Zhang, others have proposed real-time principal component analysis (PCA)-based 3D motion estimation by optimizing principal component coefficients given online imaging data.^63-67^ Extending beyond the linear correlations uncovered by PCA, it has been shown that the 2D appearance of motion during online imaging and the underlying 3D reality of internal organ motion can both be represented as manifolds, and a between-manifold mapping can be learned via CNNs to estimate 3D DVFs in real-time.^68^ Moreover, Wang et al^69^ and Shao et al^70,71^ have proposed the use of CNNs and graph CNNs that deform biomechanical meshes to model organ deformation during online imaging. Because DL approaches offer the possibility of inferring volumetric information in a single shot, they present a promising tool for the future of real-time dose-guided radiation therapy on conventional linacs. A framework for generating volumetric images from 2D projections is shown in Figure 2.

## Real-time MRI volumetric imaging

MRI is increasingly being used in radiation therapy applications because of its superior soft tissue contrast and radiation-free nature.^72^ However, MRI is also a slow imaging modality when compared with x-ray-based imaging techniques because of the time-intensive need to acquire each k-space datapoint individually and to reconstruct images with minimal latency. With current hardware, it is rarely possible to acquire and reconstruct a full 3D k-space with sufficient spatiotemporal resolution for treatment adaptation. Current MRI-guided motion-tracking strategies used to capture respiratory motion, therefore, rely on pseudo-3D, that is, interleaved sagittal/coronal 2D cine acquisitions.^73^ True 3D motion tracking with a temporal resolution of a few seconds is only applicable to slow-moving lesions such as prostate tumors.^74^

Several approaches are available to significantly accelerate the MRI acquisition, including parallel imaging techniques such as sensitivity encoding (SENSE),^75^ variable-density undersampling techniques such as compressed SENSE,^76^ or partial Fourier techniques such as half-scan.^77^ Any undersampled acquisition needs to be complemented by a reconstructor that is explicitly designed for filling in missing k-space samples, for example, in an iterative fashion, which may come with a computational penalty. Artifical intelligence (AI)-assisted reconstruction techniques can be exploited to harness spatiotemporal sparsity while featuring short inference times.^78^ DL networks have leveraged data-driven priors^79^ or geometry-driven priors^80^ for fast, volumetric MRI reconstruction of heavily undersampled acquisitions that can enable real-time tumor tracking.^81,82^ Although mostly confined to 2D imaging demonstrations, DL methods based on superresolution imaging^83^ and motion prediction^84^ have been successful in accelerating target tracking on MRI-Linacs, and it is expected that these techniques will generalize to real-time volumetric imaging in the near future.

Beyond DL methods, high levels of spatiotemporal correlation between, for example, different respiratory phases can also be exploited by non-AI methods. For example, the Model-based reconstruction of motion from undersampled signals (MR-MOTUS) framework estimates 3D DVFs directly from minimal k-space data.^85^ Real-time 3D DVFs would directly enable applications such as MLC tracking and dose accumulation, but they can also be used to reconstruct motion-corrected images. Another example is MR signature matching (MRSIGMA), which achieves real-time volumetric imaging by matching the current k-space sample with a preacquired, patient-specific 4-dimensional (4D) motion dictionary.^86^ Further developments are needed to increase the speed and robustness of real-time, volumetric MRI, especially with regard to reconstruction algorithms and online quality assurance (QA), to achieve real-time volumetric imaging. As is the case for all new imaging technologies used in radiation therapy, the integrity of dose calculations based on these real-time volumetric approaches must be rigorously evaluated before clinical use.

## Real-Time Dose Accumulation

Real-time volumetric imaging opens the door for real-time dose accumulation. Ideally, the full-time-dependent 3D patient anatomy would be known through real-time volumetric imaging and used to accumulate the patient dose in real-time during the treatment. Methods to quantify internal motion and deformations and accumulate the resulting patient dose distribution were proposed at least 25 years ago,^87^ and a quantitative analysis of normal tissue effects in the clinic (QUAN-TEC) vision paper 10 years later stressed the need for accurate dose accumulation to correlate the actually delivered dose with clinical outcome.^88^ Performing the dose accumulation in real time would allow real-time dose guidance, where decisions on treatment adaptation are based on the delivered dose, possibly combined with the potential dosimetric gain of the adaptation. Although real-time dose accumulation has not yet been performed in a real-time imaged 3D patient anatomy, it has been realized assuming either rigid tumor or organ motion or motion identical to that of a 4DCT scan.^89-91^

**Fig. 3.**
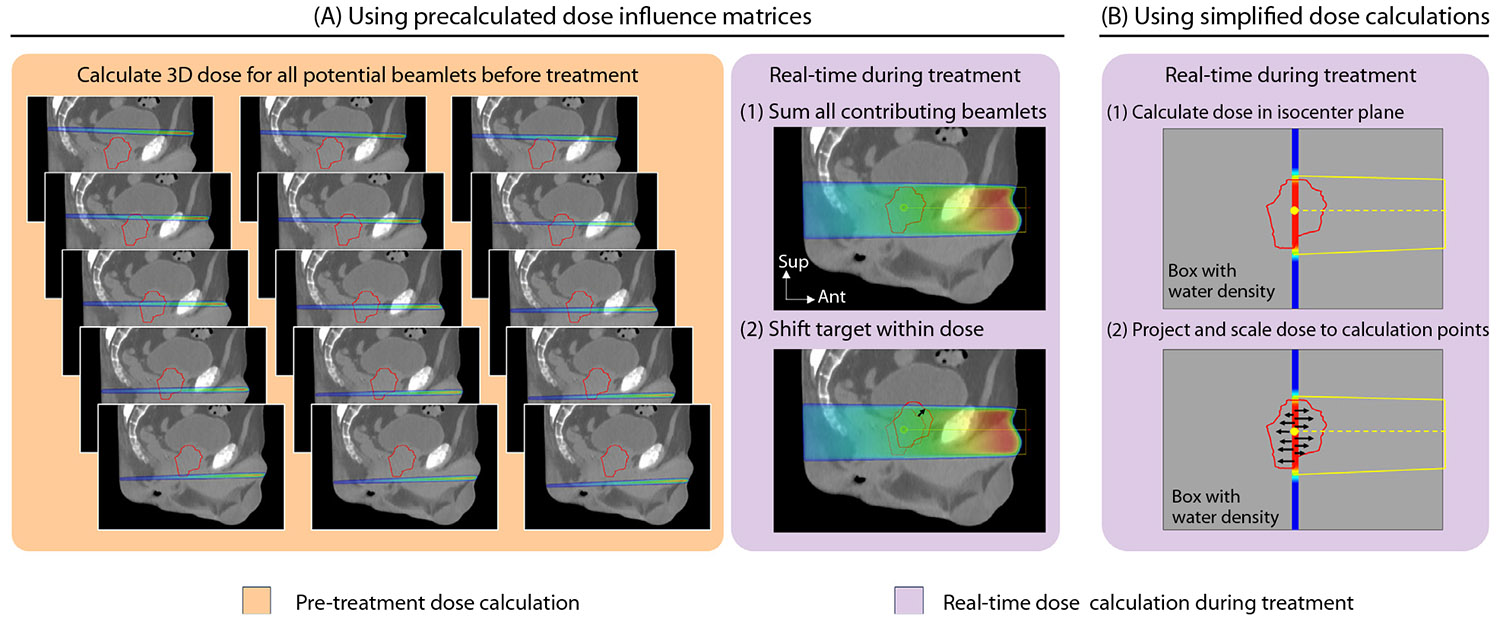
Two strategies for obtaining real-time dose accumulation speed by either using (A) precalculated dose influence matrices^89^ or (B) simplified dose calculations.^92^

Motion including dose accumulation with sufficient speed for real-time application has been obtained by either relying on precalculated doses^89^ or greatly simplified dose calculations.^92^ Fast et al^89^ used precalculated dose influence matrices that specify the patient dose from individual beamlets for real-time dose accumulation by summing contributions from all incoming beamlets per calculation time point (Fig. 3A). Target motion was modeled by sampling the dose in a shifted target volume in the patient,^89,90^ whereas dose perturbations due to respiratory anatomic changes could be included using phase-specific dose influence data from a 4DCT scan and precalculated DVFs for real-time dose mapping onto a reference phase.^90^ Limitations of this method include large libraries of precalculated doses that cannot be real-time adapted to anatomic changes not seen in the pre-treatment imaging. Ravkilde et al^92^ instead obtained real-time capability with their DoseTracker algorithm by reducing the calculation complexity. The dose was first calculated in the isocenter plane by convolving the beam aperture with a 2D dose kernel and assuming water density and a flat patient surface perpendicular to the beam axis at the planned source-surface distance. The isocenter plane dose was then projected to all calculation points in smaller or larger depths and scaled with the depth dose curve (Fig. 3B). The non-voxel-based algorithm allowed dose calculation in an arbitrary set of calculation point positions that may be shifted independently of each other between consecutive calculations to account for arbitrary motion and deformation. Although limited by assumptions of water density, a flat patient surface, and a depth-independent beam penum-bra, the algorithm has been shown to provide motion-induced dose errors with acceptable accuracy in liver^93^ and pelvis,^94^ while tissue inhomogeneity modeling would likely be needed for application in the thorax. This approach is to date the only real-time dose accumulation method that has been used online clinically.^91^ For dose accumulation applications on high-field MR-Linac systems, Monte Carlo–based dose calculation algorithms are likely needed to fully account for the electron-return effect.^95^

Dose accumulation on real-time volumetric imaging (CT or Magnetic Resonance) requires robust and fast DIR algorithms that would take into account deformable dose accumulation for real-time adaptive replanning of targets and organs. Commercial DIR methods are insufficiently accurate for aligning highly deforming and irregularly shaped organs.^96^ DIR algorithms are actively researched for radiation therapy and are offered in commercial solutions.^97-99^ AI-based DIR (or AI-DIR) methods are computationally faster and can be more accurate than standard DIR methods because of their ability to directly predict deformations given 2 images.^96,100-102^

**Fig. 4.**
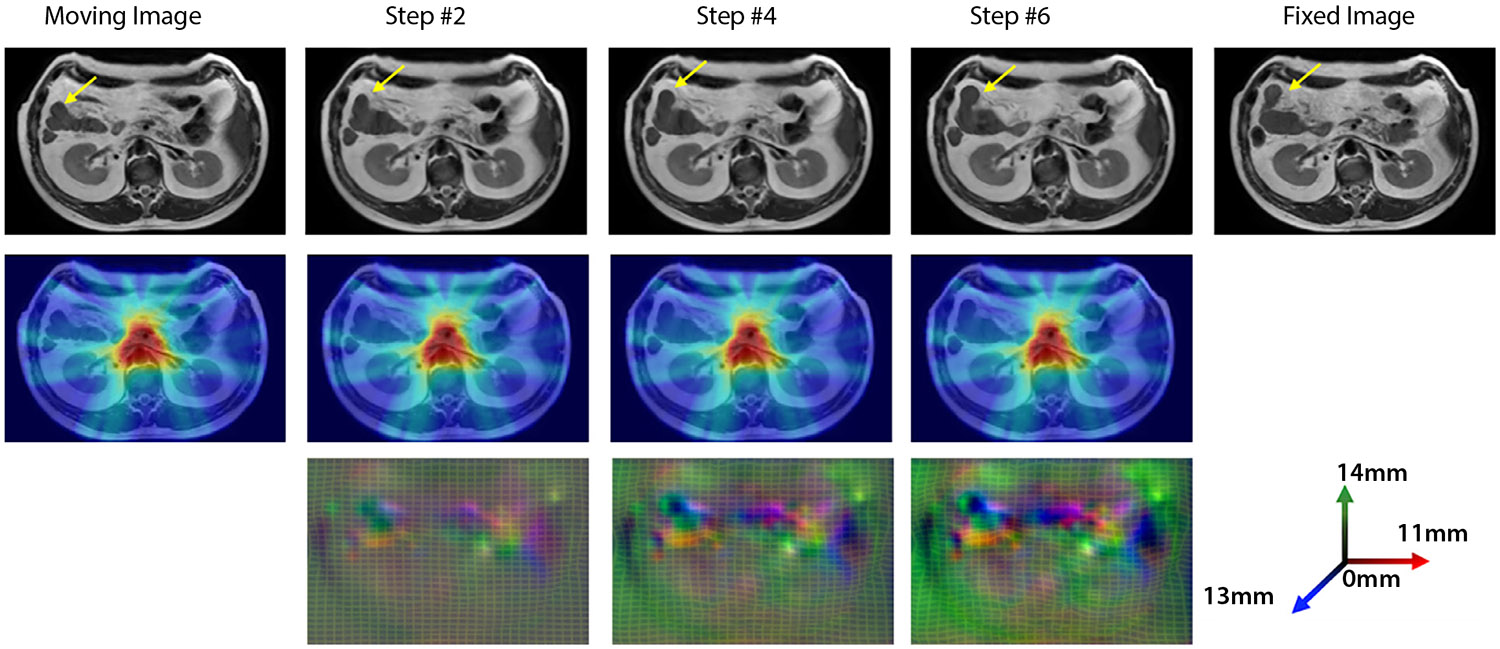
A time series of anatomic deformation, dose accumulation, and deformation vector fields between a moving and a fixed image performed using progressively refined registration segmentation AI-DIR method. The complex deformation of the anatomy is highlighted by the yellow arrows.

However, predominant AI-DIR and DIR methods use small deformation frameworks, which cannot preserve topology in the presence of large deformations.^103,104^ DIR methods, such as the large diffeomorphic metric mapping, are more accurate because they estimate the diffeomorphic (topology preserving) deformations by integrating a time-varying velocity field using computationally expensive high-dimensional optimization. This method has been used with modifications including multistage and multiobjective constraints for intensity and organ geometry for complex gastrointestinal organ deformation.^105^ Even though DIR methods could be extremely challenging for interpatient or even interfraction anatomy, they could be quite robust to intrafraction changes.^106^ Jiang et al^107^ have proposed a fast AI-DIR method called progressively refined registration segmentation, which robustly handles large deformations by progressively refining local alignment with time-varying dense deformation flows to allow spatially and temporally varying regularization. This network is constructed using a 3D convolutional long short-term memory network and dense flow field of voxel-wise deformations. The convolutional long short-term memory is implemented into the encoders of the registration (3D U-net) and a jointly trained segmentation (3D U-net architecture) network to model the sequential dynamics. Instead of computing warped images after each recurrent step, the DVF was directly input to the next step. Figure 4 shows a series of intermediate deformations and accumulated doses between a moving and a fixed image.

Deformable dose accumulation is typically performed by warping the dose grids after appropriate resampling of the dose grid to image resolution followed by scaling of the warped and voxel-wise dose summation, a technique known as direct dose mapping. Another approach for dose summation uses energy/mass transfer (EMT) mapping^108^ where the dose to each voxel is computed by the ratio of the deposited energy (dose × mass) and the mass (density × volume) of each voxel. Unlike direct dose mapping, the EMT mapping approach ensures the conservation of mass and energy. EMT requires tissue density information for mass recalculation. A real-time EMT implementation was demonstrated for online 4D dose accumulation.^109^ For an MRI data set, a synthetic CT would need to be generated which can also be used for calculating the electron-return effect in an integrated MRI-guided radiotherapy system.^95^

## Real-Time Volumetric Dose Measurement

The ability to visualize radiation delivery in real-time (in vivo dosimetry) poses a major limitation on real-time dose adaptation in comparison with offline applications. This is important to account for varying organ motion challenges, reducing margins, and limiting side effects to uninvolved normal tissue. Previous technologies such as inserted metal-oxide semiconductor field-effect transistor (MOSFET) detectors failed to support a wide range of in vivo dosimetry applications because of their invasive nature.

**Fig. 5.**
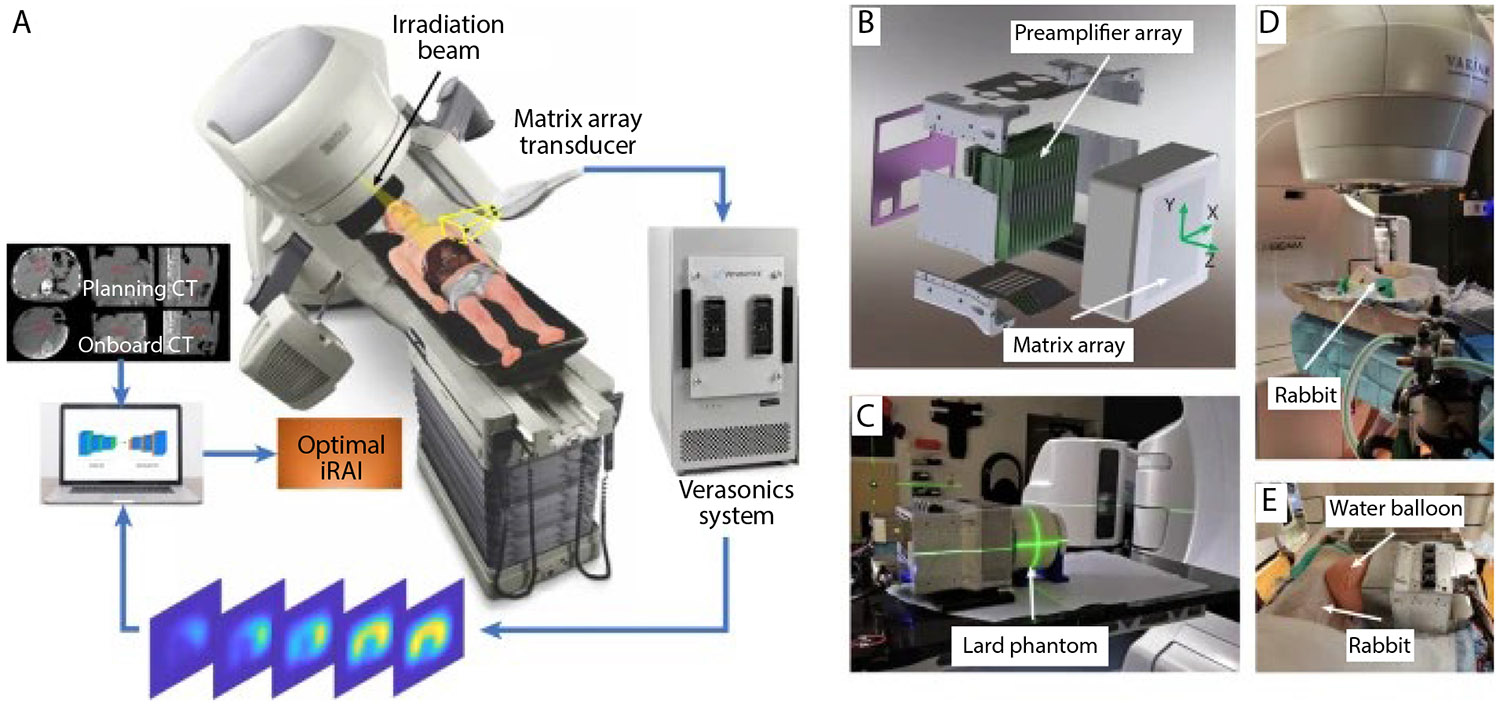
Schematic of ionizing radiation-induced acoustic imaging (iRAI) system and the experimental setup. A, Three-dimensional schematic of the iRAI system for mapping the dose deposition in a patient during radiation therapy delivery. B, Computer-aided design view of a 2-dimensional matrix array with an integrated preamplifier board. The coordinate system for the 3-dimensional iRAI imaging space is marked. C, The experimental setup for the phantom studies. D, The side view of the rabbit experiment setup in a clinical environment. E, Details regarding the transducer position and coupling of the rabbit experiment. From ref.^16^

**Fig. 6.**
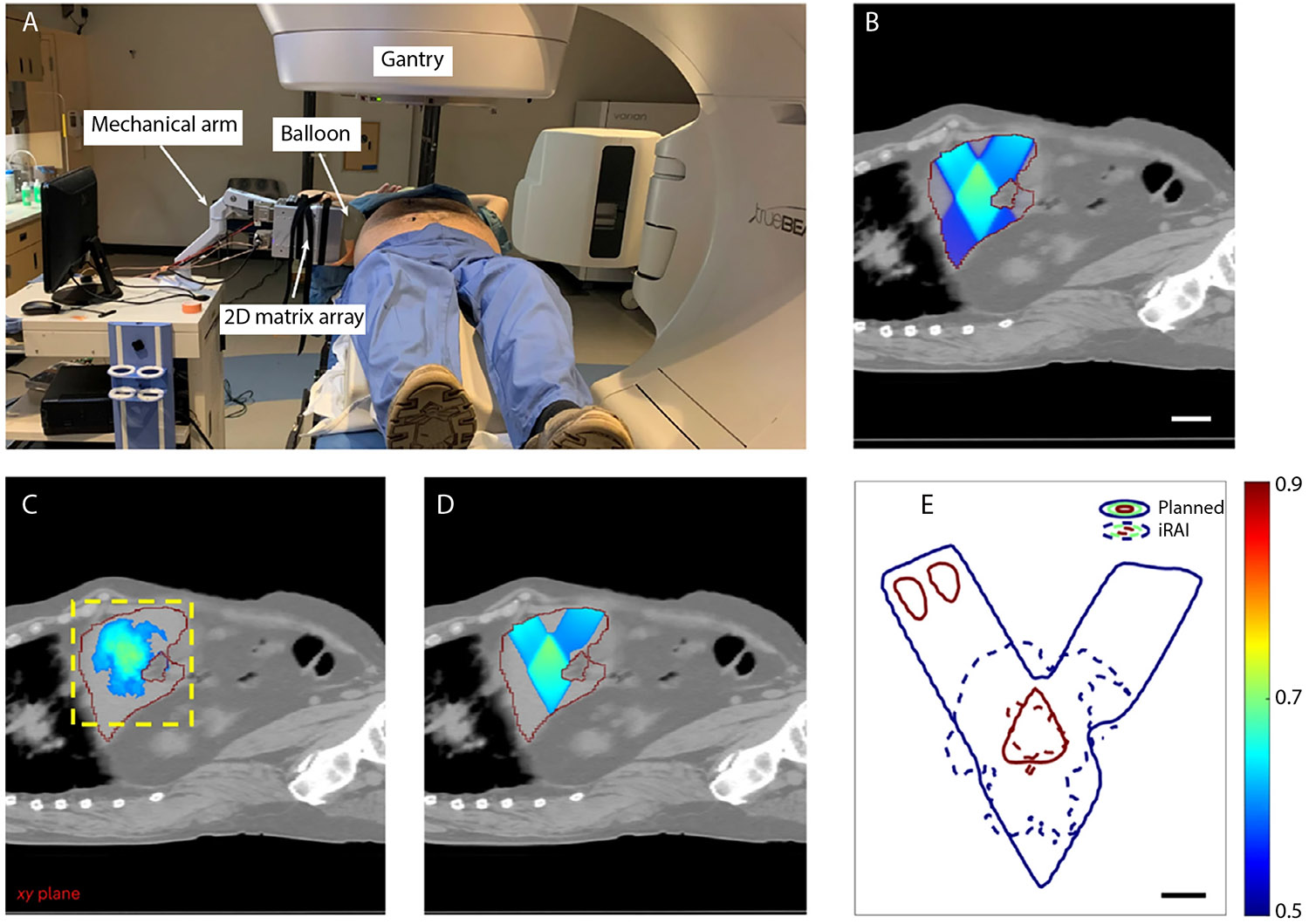
In vivo ionizing radiation-induced acoustic imaging (iRAI) versus the treatment plan on a patient. A, A photograph of the iRAI imaging on a patient taken during radiation therapy. B, The dose distribution of only the 2 static sagittal beams of the treatment plan with a liver mask fused onto the computed tomography scan anatomy structure. Scale bar, 5 cm. C, The iRAI measurement of dose with a liver mask fused onto the computed tomography anatomy structure with the same position as b. The yellow dashed box indicates the field of view of the 2-dimensional matrix array. D, Dose distribution (>50%) of the treatment plan with a liver mask fused on the computed tomography anatomy structure. E, The 50% and 90% isodose lines in the iRAI measurement and the treatment plan. Scale bar, 2 cm. The red line in B to D indicates the boundary of the liver. From ref.^16^

Recently, techniques based on optical imaging such as scintillation or Cherenkov imaging have been demonstrated to overcome these challenges. However, they are limited to surface measurements and do not allow for any real-time monitoring of dose in deeper tissue. Alternatively, ionizing radiation-induced acoustic imaging (iRAI) can be used to image in vivo radiation delivery. iRAI is based on the known thermoacoustic phenomenon in radiation physics, where acoustic waves are generated from the thermoelastic expansion of a substance after absorption of a penetrating high-energy megavoltage (MV) radiation beam.^110^ One experimental system includes a matrix array transducer to enable 3D image acquisition. The raw signal from the transducer is processed using a commercial ultrasound system (Verason-ics) (Fig. 5). The feasibility was demonstrated for 3D real-time dose mapping in simulations, physical phantoms, pre-clinical rabbit models, and a pilot clinical study,^16^ as shown in Figure 6. This system was also integrated with ultrasound B-mode imaging providing dual iRAI/ultrasound imaging capabilities. Another natural extension is in ultra high-dose rate radiotherapy, where it can benefit from the higher signal that it generates.^111^

Most C-arm linacs also provide access to electronic portal imaging devices (EPIDs) that can be used for in vivo dosime-try.^112^ For instance, Li et al^113^ explored dose-guided radiation therapy using EPID-based 3D in vivo verification. RapidArc therapy leverages in vivo dosimetry using an EPID cassette to evaluate the dose.^114^ EPID dosimetry has been used in MRI-guided treatments to detect patient motion and workflow incidents.^115^ Moreover, large-scale analyses of thousands of patients have suggested the effectiveness of EPID-based dosimetry for patient-specific dose verification.^116,117^

## Real-Time Dose-Guided Treatment Adaptation

**Fig. 7.**
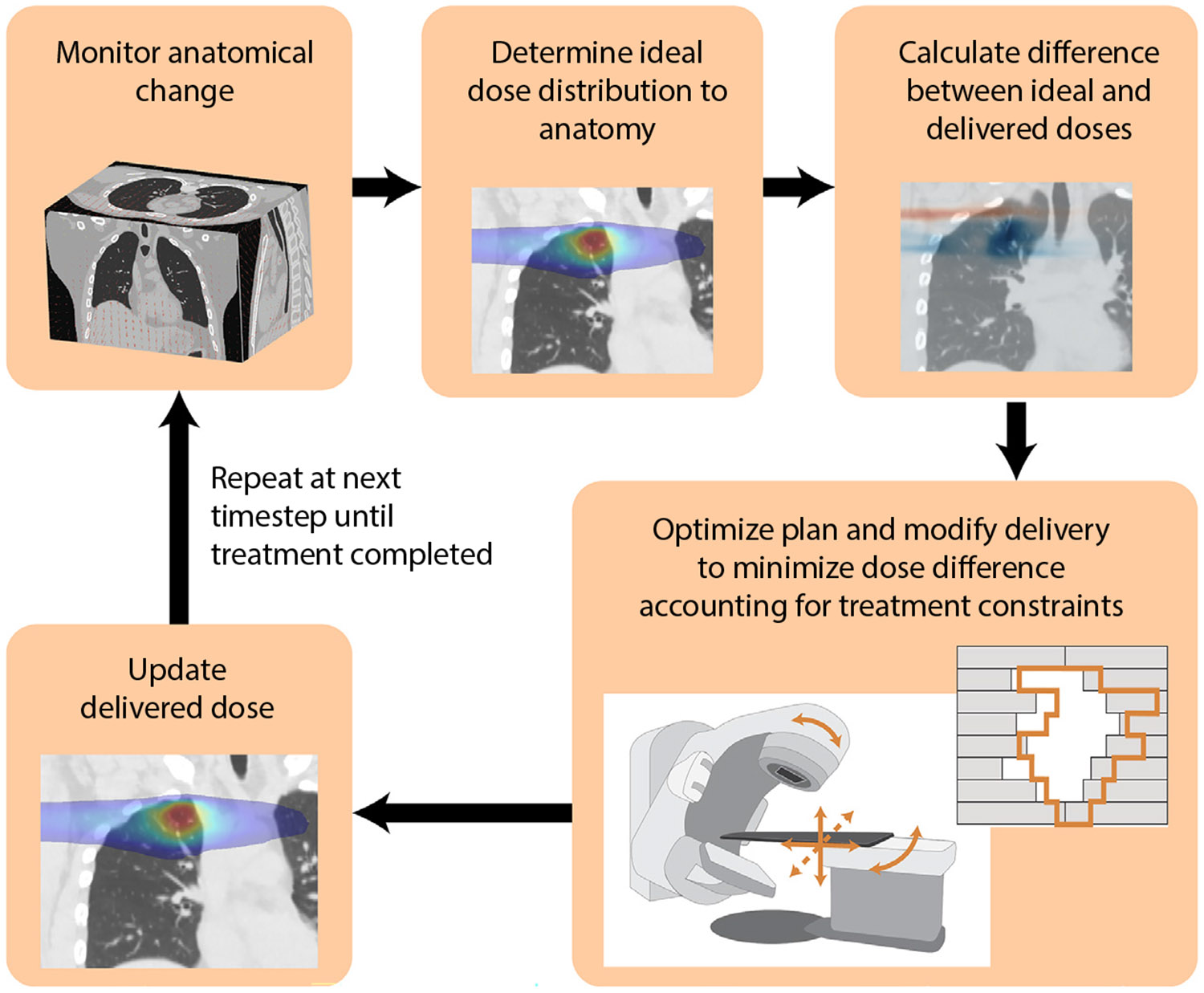
An overview of the continuous process occurring during real-time dose-guided radiation therapy optimized to the delivered dose.

With the ongoing development of motion monitoring techniques that advance beyond rigid, single-target translations to provide real-time volumetric and dosimetric information, it follows on that treatment techniques should use this additional information to adapt in response to gain the benefits of monitoring these anatomic changes. Previously the main intrafraction motion mitigation strategies have included gating or rigid realignments of the patient or beam such as through couch repositioning,^6^ trailing,^118^ MLC tracking,^28^ or robotic and gimbaled linacs.^119,120^ Real-time dose-guided radiation therapy can instead improve on these strategies by responding to dosimetric variations resulting from intrafraction anatomic changes by modifying plan parameters including MLC leaf positions, gantry angle, or collimator angle during treatment delivery to maximize the dose to the tumor and minimize the dose to the healthy tissue in the presence of ever-evolving anatomy (Fig. 7).

Kontaxis et al^121,122^ began to explore the concept of intrafraction replanning for intensity modulated radiation therapy treatments performed on an MRI-Linac. During the intervals between beam deliveries, a fluence optimization was performed to minimize the difference between a delivered and ideal dose based on the dose that had been accumulated to anatomic changes observed on 2D cine MRI. Gerlach et al^123^ investigated a beam reoptimization strategy for robotic SABR that reweighted beams from an original plan based on doses delivered to a target undergoing differential motion to OARs. Their intrafraction replanning method occurred between beam deliveries and reported improvements in dose coverage to the targets from 88% to 95% compared with performing rigid translational beam corrections while limiting overdosage to OARs. Muurholm et al^124^ described a dose-guided couch correction strategy that involved continuously predicting the final dose that would be delivered to a moving tumor using the current couch position as well as with a dose-optimized couch position to determine whether a repositioning of the couch would be beneficial for the dose delivered to the clinical target volume.

Continuous, real-time dose-guided MLC reoptimization for volumetric modulated arc therapy treatments was first demonstrated by Mejnertsen et al^125^ This method performed fast 3D dose calculations which were reduced to 2D in the beam’s-eye-view plane and each MLC leaf pair position was optimized based on minimizing the difference between the planned and delivered doses along each leaf track at each time point. The method was applied in silico to a prostate treatment data set and showed a reduction in gamma-failure rates between the planned and delivered doses compared with conventional rigid MLC tracking.^125^ Dose-guided adaptation was extended to anatomy with differential motion by Hewson et al^126^ where motions of the prostate and pelvic lymph nodes were simultaneously adapted by optimizing the MLC leaf positions to doses accumulated in separate dose clouds for each target of interest. Initial work toward reoptimizing MLC apertures to the entire deforming anatomy has also been investigated by considering deformable dose grids for multiple lesions within the lung.^127^

## Discussion

This review has surveyed the emerging technologies for real-time dose-guided radiation therapy, describing the expected clinical impact of the technology, along with developments in the 3 core process steps: real-time volumetric imaging, dose accumulation, and dose-guided treatment adaptation. The review integrates the 3 steps that are typically studied separately. Future treatment devices will need all 3 process steps integrated to achieve the clinical benefit of tumor targeting and normal tissue avoidance offered by real-time dose-guided radiation therapy.

The advances are primarily software-driven and automated upgrades that can build on existing cancer treatment hardware. Automated developments can increase treatment efficiency. For example, a clinical trial of MLC-tracking real-time 3D IGRT was stated to be “feasible, efficient and deliver high-precision target dose and lower normal tissue dose,”^28^ demonstrating that software solutions can also be widely available and cost-effective.

The bulk of radiation therapy systems used globally have onboard kilovoltage x-ray imaging. Therefore, dose-guided radiation therapy advances that can be implemented on linacs with x-ray imagers have the potential for immediate and wide-spread application without the need for expensive, dedicated systems. Such low-cost options are vital when, for instance, a majority of radiation therapy centers wish to offer real-time motion management to more patients but are hindered by financial and human resources.^128^ On the other hand, MRI offers high soft tissue contrast without radiation exposure with the downside of slower acquisition speed. As a result, current MRI-guided gating and MLC tracking often rely on interleaved 2D cine acquisitions, which are limited to capturing a single target and often do not provide OAR information. To address these limitations, various acceleration strategies are deployed to achieve truly real-time, volumetric MRI: parallel imaging and undersampling techniques, AI-assisted reconstructions, and model-based approaches. Looking forward, continued improvements in speed, robustness, and real-time QA for reconstruction algorithms are essential to make both x-ray- and MRI-based volumetric imaging a viable tool for adaptive radiation therapy. The ultimate goal is to achieve real-time, high-resolution strategies to provide reliable inputs for motion-tracking algorithms and real-time dose accumulation.

Real-time dose accumulation is still at an early stage with only one report on online clinical application for rigidly moving single targets in an observational study without any dose-guided treatment adaptations.^91^ The gain of dose-guided treatment adaptations compared with geometric guidance will become much more important for deforming and differentially moving multiple targets or OARs, where rigid motion assumptions are insufficient to inform the adaptation. The relevance of real-time dose accumulation is therefore expected to increase significantly with further developments in real-time 3D imaging or other means to assess differential motion in real time. If the dose-guided treatment adaptations are not performed continuously in real time as in MLC tracking, but rather as time-consuming treatment interruptions and corrections in case of unacceptable doses, then the dose accumulation should be supplemented with real-time predictions of the final fraction dose^124^ to provide full dose-volume histograms for decision-making.

When considering DIR-based dose accumulation, practical and robust guidelines are needed to assess uncertainties in dose accumulation during the online adaptive process. For QA of DIR warping quantitative information, a recent European Society for Radiotherapy and Oncology workshop summary recommends at least a visual inspection of image similarity and potentially DVF for alignment. For plausibility, at least a voxel-wise Jacobian determinant and/or bending energy and dose warping specifically, visualizing the distance to dose difference or dose gradient is necessary.^129^ In the context of real-time dose accumulation, visual inspection is not possible, and automated approaches need to be relied upon. This reliance may require improved commissioning, posttreatment assessment, and the need for automated processes to report confidence in the tasks being performed.

Although real-time treatment adaptation methods that take advantage of volumetric and dosimetric information have begun to emerge, these methods have primarily been evaluated through retrospective treatment analyses and have yet to be clinically implemented. Future real-time treatment adaptation methods could also consider exploiting all the available degrees of freedom on a treatment system, including reoptimizing collimator and gantry angles, couch position, and beam energies to adapt to the anatomy observed during treatment. Current real-time dose-guided MLC optimization strategies also require an initial treatment plan with a dose distribution that the MLC optimization will aim to reproduce.^125,126^ However, future methods could aim to deliver a dose distribution that is superior to the original treatment plan if a more favorable anatomic geometry is seen during treatment. Other types of variations in the patient such as functional or biological changes could also be incorporated into the plan reoptimization strategies.

Artificial intelligence has already demonstrated the significant potential for time savings in prospective radiation treatment planning studies.^130^ Arguably, the deployment of AI for real-time radiation therapy will be even more transformative by enabling imaging and treatment beam adaptations with extremely low latencies that are not currently achievable. AI solutions naturally align with real-time imaging and treatment adaptation problems because the computationally expensive learning phase can be performed offline, with the inference operations used during real-time deployment being computationally efficient and compatible with real-time deployment.^131,132^ To solve the ill-posed problem of estimating 3D images or motion from sparse 2D imaging data, a latent low-dimensional representation of the output space can be discovered in a patient-specific manner.^57,68^ These approaches typically train DL models using planning data; however, there may be significant anatomic interfraction variation that may require additional fine-tuning on the treatment day. Moreover, such patient-specific methods will require novel QA before prospective trials can be performed.^133^ Increasingly, AI-based methods for subsecond 4D MRI reconstruction are being developed to improve real-time volumetric imaging on MRI-Linacs.^134,135^ Recently, generalizable segmentation models such as nnUnet have been applied to cardiac segmentation for substructure segmentation during stereotactic ablation treatments for ventricular tachycardia; however, further improvements in inference time will be needed for real-time adaptation.^136,137^

The focus of this review is external beam x-ray radiation therapy. For brachytherapy^138^ and particle therapy,^139^ the same principles could be applied for real-time anatomy adaptation. However, the equipment and constraints of the three core process steps of volumetric imaging, dose accumulation, and real-time dose-guided treatment adaptation would vary.

With real-time 3D IGRT showing higher dose to tumors,^7,8^ and halving of toxicity compared with standard treatments,^2,3^ there is a tremendous potential for real-time dose-guided radiation therapy to further improve patient outcomes, offering future cancer patients an accessible, efficient, cost-effective treatment modality.

To temper the argument for increased technological development in dose-guided radiation therapy one must ask, is there a point of diminishing return for dose conformality? Where does dose-guided radiation therapy fit within the broader context of future technology of radiation therapy? Although there are many other uncertainties, particularly target delineation, the known response of normal tissue toxicity with dose, highlighted in the QUANTEC and hypofractionated treatment effects in the clinic (HYTEC) special issues of this journal, we can posit that decreases in dose will reduce toxicity and therefore improve the quality of life, although the magnitude of the benefit is difficult to quantify. The increased targeting with dose-guided radiation therapy may open up opportunities for routine knowledge of dose-response tissues, such as neurovascular bundle, heart substructure, and total blood sparing. Dose-guided radiation therapy may continue to enable more patients to have access to hypofractionation, and the patient, and health system cost benefits of fewer fraction treatments. The decreasing cost-effectiveness of diminishing returns may count against continued technology development and also move research and development to other emerging areas of radiation therapy, such as immunotherapy-radiation therapy interactions.

## Conclusion

The emerging field of real-time dose-guided radiation therapy has the potential to significantly improve patient outcomes. The advances are primarily software-driven and therefore could be widely available and cost-effective upgrades to improve imaging and targeting cancer.
